# Surgery for Locally Advanced Pancreatic Cancer Following Induction Chemotherapy: A Single-Center Experience

**DOI:** 10.1245/s10434-024-15591-4

**Published:** 2024-07-02

**Authors:** Rutger T. Theijse, Thomas F. Stoop, Philip D. Leenart, Kishan R. D. Lutchman, Joris I. Erdmann, Freek Daams, Babs M. Zonderhuis, Sebastiaan Festen, Rutger-Jan Swijnenburg, Thomas M. van Gulik, Annuska Schoorlemmer, André L. A. Sterk, Susan van Dieren, Arantza Fariña, Rogier P. Voermans, Johanna W. Wilmink, Geert Kazemier, Olivier R. Busch, Marc G. Besselink, For HPB-Amsterdam

**Affiliations:** 1 Department of Surgery, Amsterdam UMC, Location University of Amsterdam, 1081 HV Amsterdam, The Netherlands; 2 Cancer Center Amsterdam, 1081 HV Amsterdam, The Netherlands; 3 Department of Surgery, Amsterdam UMC, Location Vrije Universiteit, Amsterdam, The Netherlands; 4 Department of Surgery, OLVG, Amsterdam, The Netherlands; 5 Department of Pathology, Amsterdam UMC, Location University of Amsterdam, Amsterdam, The Netherlands; 6 Department of Gastroenterology and Hepatology, Amsterdam UMC, Location University of Amsterdam, Amsterdam, The Netherlands; 7 Amsterdam Gastroenterology Endocrinology Metabolism, Amsterdam, The Netherlands; 8 Department of Medical Oncology, Amsterdam UMC, Location University of Amsterdam, Amsterdam, The Netherlands

**Keywords:** Pancreatic cancer, Locally advanced, Induction therapy, Resection, Surgical exploration

## Abstract

**Background:**

The use of surgery in patients with locally advanced pancreatic cancer (LAPC) following induction chemotherapy is increasing. However, most series do not report on the total cohort of patients undergoing surgical exploration; therefore, this single-center study investigates outcomes among all consecutive patients with LAPC who underwent surgical exploration.

**Methods:**

We conducted a retrospective, single-center analysis including all consecutive patients with LAPC (Dutch Pancreatic Cancer Group criteria) who underwent surgical exploration with curative intent (January 2014–June 2023) after induction therapy. Primary outcomes were resection rate and overall survival (OS) from the time of diagnosis.

**Results:**

Overall, 127 patients underwent surgical exploration for LAPC, whereby 100 patients (78.7%) underwent resection and 27 patients (21.3%) underwent a non-therapeutic laparotomy due to the extent of vascular involvement (*n* = 11, 8.7%) or occult metastases (*n* = 16, 12.6%). The overall in-hospital/30-day mortality rate was 0.8% and major morbidity was 31.3% (in patients after resection: 1.0% and 33.3%, respectively). The overall 90-day mortality rate was 5.5%, which included 3.1% mortality due to disease progression. Resection was associated with longer median OS {29 months (95% confidence interval [CI] 26–43) vs. 17 months (95% CI 11–26)*; p *< 0.001} compared with patients undergoing non-therapeutic laparotomy, with corresponding 5-year OS rates of 28.4% and 7.7%. In Cox proportional hazard regression analysis, only pancreatic body/tail tumors independently predicted OS (hazard ratio 1.788 [95% CI 1.042–3.068]).

**Conclusion:**

This single-center series found a resection rate of 78.7% in patients with LAPC selected for surgical exploration, with a low risk of mortality and morbidity in all explored patients and a 5-year OS rate after resection of 28.4%.

**Supplementary Information:**

The online version contains supplementary material available at 10.1245/s10434-024-15591-4.

Approximately 30–40% of patients with pancreatic adenocarcinoma are diagnosed with locally advanced pancreatic cancer (LAPC) due to extensive vascular tumor involvement. Upfront surgery in these patients is precluded because of high surgical risks in the absence of survival benefit;^1,2^ therefore, patients with LAPC have historically been treated with non-curative intent, comprising either palliative chemotherapy or best supportive care.

Over the last decade, the treatment for LAPC strongly evolved due to the introduction of novel multiagent chemotherapy regimens (i.e., [m]FOLFIRINOX and gemcitabine-nab-paclitaxel), which are associated with improved disease control, resection rates, and increased overall survival (OS).^3,4^ International expert centers have gradually improved their selection process for surgical resection of LAPC,^5^ using anatomical, biological and conditional parameters.^6,7^ As a consequence, approximately 19–22% of selected patients with LAPC undergo resection, associated with a median OS of 28–33 months.^8,9^

Regardless of improved induction therapy regimens and adequate response evaluation, a considerable subgroup of surgically explored patients with LAPC will undergo a non-therapeutic laparotomy without resection, due to either extensive local involvement or radiological occult metastases.^10^ However, most series do not report the rate of non-therapeutic laparotomy in this setting, let alone the postoperative outcome in this subgroup.^11–17^ Moreover, studies reporting on predictors for surgical and oncological outcomes in a cohort of explored LAPC patients within an homogenous, high-volume center, which may guide and optimize patient selection for surgery, are currently lacking.

Therefore, this study sought to investigate postoperative outcome and predictors for OS among all consecutive patients with LAPC who underwent surgical exploration with intention for resection in a single high-volume center in The Netherlands.

## Methods

### Study Design

This single-center, retrospective study was performed following the Strengthening the Reporting of Observational Studies in Epidemiology (STROBE) guidelines.^18^ Data were extracted from the mandatory prospective Dutch Pancreatic Cancer Audit,^19^ whereby additional data were obtained through the prospectively maintained nationwide LAPC Registry.^20^ Missing data were collected from local electronic medical records.

### Study Population

All consecutive patients (≥18 years of age) with LAPC at diagnosis who underwent a surgical exploration with intention for a resection following systemic induction chemotherapy in the Amsterdam UMC were included (1 January 2014 to 30 June 2023). Patients with (borderline) resectable disease who progressed to LAPC during neoadjuvant chemoradiotherapy were also included, as long as they thereafter received a second-line induction therapy for LAPC.

### Outcomes

The primary outcome was resection rate and OS from the time of diagnosis (i.e., imaging-based and pathology confirmed). Secondary outcomes included pre- and intraoperative predictors for OS in the overall cohort as well as in-hospital/30-day mortality and major morbidity, and 90-day mortality.

### Definitions

LAPC was defined in accordance with the Dutch Pancreatic Cancer Group (DPCG) criteria: >90° arterial involvement (i.e., superior mesenteric artery, celiac axis, and/or any hepatic artery) and/or >270° portomesenteric venous involvement.^21^ In the case of occlusion or non-reconstructable portomesenteric venous involvement, patients were also classified as LAPC, as outlined in electronic supplementary Table 1.^21^ Additionally, LAPC was also defined in accordance with the 2022 National Comprehensive Cancer Network (NCCN) guideline.^22^ Further details regarding methodological definitions are described in Appendix 1.

### Patient Selection

From January 2014 to May 2021, surgical procedures were performed at location AMC of Amsterdam UMC. Thereafter, following a hospital merger, all procedures were performed at location VUmc of Amsterdam UMC (June 2021–June 2023). In general, patients with non-progressive disease (Response Evaluation Criteria in Solid Tumors [RECIST] criteria)^23^ on four-phase computed tomography (CT) scan combined with serum tumor marker response following at least 2 months of systemic chemotherapy (either four cycles of [m]FOLFIRINOX or two cycles of gemcitabine-nab-paclitaxel) were selected for surgery during a multidisciplinary team meeting. Serum carbohydrate antigen 19-9 (CA19-9) and, since 2021, fluorodeoxyglucose positron emission tomography with CT (FDG-PET/CT) were used to determine the biological tumor response to induction therapy.^24^ In January 2022, the nationwide PREOPANC-4 trial started in the Amsterdam UMC (ClinicalTrials.gov identifier: NCT05524090), wherein the international best-practice for LAPC care is implemented in The Netherlands, in close collaboration with four international experts.^25^ As a consequence, the institutional treatment protocol for patients with LAPC changed. First, the induction phase was extended from at least 2 months to a minimum of 4 months of chemotherapy (i.e., eight cycles [m]FOLFIRINOX/four cycles gemcitabine-nab-paclitaxel), whereby a chemotherapy switch (i.e., second-line therapy) was considered in case of biological and/or radiological (local) disease progression.

### Statistical Analyses

Data analyses were performed using RStudio: Integrated Development Environment for R (software version 1.3.1093, Boston, MA, USA).^26^ Descriptive statistics were compiled to summarize patient characteristics. Pearson’s Chi-square, or Fisher’s exact test when appropriate, was used to compare categorical variables, and the Mann–Whitney *U* test was used to compare numerical variables. The Cochran–Armitage Test was used to assess trends over time. Categorical variables are presented as percentages and frequencies, and numerical variables as medians with corresponding interquartile ranges (IQRs). For all tests, statistical significance was defined as a two-tailed *p*-value of <0.050. Recurrence-free survival was calculated from the date of surgery, and OS was calculated from the date of diagnosis to the date of death or last follow-up, and presented using Kaplan–Meier estimates with their corresponding 95% confidence intervals (CIs). Both were assessed using the log-rank test. Patients still alive during the date of final follow-up (31 October 2023) were censored. Median follow-up was calculated through reversed Kaplan–Meier estimates.

Cox proportional hazards regression analysis was performed to identify potential predictors for OS. Results of the regression analysis are presented as hazard ratios (HR) with corresponding 95% CIs. Clinical predictors with a *p*-value <0.200 in univariable analysis were included in the multivariable analysis. Backwards stepwise selection was used for the removal of insignificant variables in multivariable analysis, until all remaining variables were statistically significant.^27^ In order to correct for implementation of the international best practice for LAPC in our center (including the training program from 2021 to 2022), a categorical variable was added in multivariable analysis distinguishing patients treated between 2014–2020 and 2021–2023. The relative serum CA19-9 response following induction therapy was categorized according to the optimal response cut-off value for LAPC, as determined previously.^28^

Preferably, imputation was performed for the missing CA19-9 data. However, a broad variety existed between the imputed datasets and the original dataset that could have resulted in misinterpretation of outcomes; therefore, no imputation was performed and an additional ‘missing’ category was added for each categorical variable that was missing ≥2%. Patients with missing data for variables missing <2% were excluded. If the only significant level in a variable was the ‘missing’ category, this independent variable was not included in the multivariable analysis.

## Results

During the study period, 127 patients with LAPC were selected for surgical exploration with intention for resection and were subsequently included in this study. All patients received systemic induction chemotherapy, which mostly consisted of (m)FOLFIRINOX (*n* = 117, 92.1%) with a median of four cycles (4–8), whereby eight patients (6.3%) were treated with second-line induction chemotherapy prior to surgery. In total, four patients (3.1%) who were initially diagnosed with borderline resectable disease who progressed to LAPC during neoadjuvant therapy were included (see Table 1 for patient and treatment characteristics).

**Table 1:** Baseline characteristics of 127 patients with locally advanced pancreatic cancer undergoing surgical exploration

		Total cohort		
Characteristics	Overall [*n *= 127]	Resection [*n *= 100]	Non-therapeutic laparotomy [*n *= 27]	*p*-Value
Sex, male, *n *(%)	67 (52.8)	51 (51.0)	16 (59.3)	0.446^a^
Age, years, median [IQR]	64 [58–70]	64 [57–70]	66 [61–71]	0.200^b^
ECOG PS ≥2, *n *(%)	8 (6.5)	6 (6.0)	2 (7.4)	0.999^c^
BMI, kg/m^2^, median [IQR]	24 [22–26]	24 [22–26]	23 [21–26]	0.798^b^
Location	–	–	–	0.049^a^
Head, *n* (%)	94 (74.0)	78 (78.0)	16.0 (59.3)	–
Body/tail, *n* (%)	33 (26.0)	22 (22.0)	11.0 (40.7)	–
NCCN LAPC, *n* (%)	49 (38.6)	36 (36.0)	13 (48.1)	0.250

Disease characteristics at diagnosis				
Tumor size, mm, median [IQR]	37 [30–45]	37 [29–44]	40 (34–46)	0.372^b^
Missing	14	11	3	–
CA19-9, median [IQR]^$^	268 [49–690]	286 [50–766]	226 (35–363)	0.275^b^
<37 U/mL (normal)	24 (22.4)	18 (21.2)	6 (27.3)	0.501^c^
≥37 to <150 U/mL	15 (14.0)	13 (15.3)	2 (9.1)	–
≥150 to <500 U/mL	33 (30.8)	24 (28.2)	9 (40.9)	–
≥500 U/mL	35 (32.7)	30 (35.3)	5 (22.7)	–
Missing	20	15	5	–
CEA, median [IQR]^$^	4 [3–6]	4 [3–6]	4 (3–6)	0.772^b^
Normal (≤5 ng/mL), *n *(%)	42 (67.7)	37 (67.3)	5 (71.4)	0.999^c^
Elevated (>5 ng/mL), *n *(%)	20 (32.3)	18 (32.7)	2 (28.6)	–
Missing	65	45	20	–

Treatment details				
First-line induction chemotherapy, *n* (%)	–	–	–	0.200^c^
(m)FOLFIRINOX	117 (92.1)	94 (94.0)	23 (85.2)	–
Gemcitabine-nab-paclitaxel	4 (3.1)	3 (3.0)	1 (3.7)	–
Other multiagent regimens	2 (1.6)	1 (1.0)	1 (3.7)	–
Gemcitabine	4 (3.1)	2 (2.0)	2 (7.4)	–
Number of cycles, (median [IQR])	4 [4–7]	4 [4–7]	5 [4–8]	0.526^b^
(m)FOLFIRINOX cycles	4 [4–8]	4 [4–7]	5 [4–8]	0.371^b^
Second-line induction therapy, *n *(%)	8 (6.3)	7 (6.9)	1 (3.7)	0.999^c^
(m)FOLFIRINOX	2 (25.0)	2 (28.6)	0 (0.0)	–
Gemcitabine-nab-paclitaxel	5 (62.5)	4 (57.1)	1 (100.0)	–
Capecitabine	1 (12.5)	1 (14.3)	0 (0.0)	–
Time diagnosis to surgery, days, median [IQR]	155 (127–196)	154 [128–195]	159 [127–198]	0.554^b^

*n* Number of patients, *IQR* Interquartile range, *ECOG PS* Eastern Cooperative Oncology Group performance status, *BMI* Body mass index, *NCCN* National Comprehensive Cancer Network, *LAPC* Locally advanced pancreatic cancer, *CA19-9* Carbohydrate antigen 19-9, *CEA* Carcinoembryonic antigen

^*a*^Pearson Chi square test; ^*b*^Mann-Whitney *U* test; ^*c*^Fisher’s exact test

### Response to Induction Therapy

The majority of patients (*n* = 78, 75.7%) presented with RECIST stable disease on imaging following systemic induction therapy, while the remaining patients presented with either partial response (*n* = 24, 23.3%) or local progressive disease (*n* = 1, 1.0%) at restaging. This latter patient arbitrarily underwent surgery because of stable non-elevated serum CA19-9 during chemotherapy and no change in vascular involvement, although tumor size increased.

At restaging, the median serum CA19-9 level was 62 U/mL (IQR 23–186), including normalization in 17 patients (22.1%) among those with elevated CA19-9 prior to induction therapy. No significant differences were observed in CA19-9 response to induction therapy between those resected and those undergoing non-therapeutic laparotomy. However, patients not undergoing resection more often had non-elevated (<37 U/mL) serum CA19-9 levels at restaging (30.4% vs. 51.9%; *p *= 0.004) [see electronic supplementary Table 2 for anatomical and biological tumor response to induction therapy].

### Surgical Procedures and Outcome

Diagnostic laparoscopy was performed in 101 patients (79.9%) at the start of surgical exploration, whereby an annual increase was seen from 16.7% in 2014 to 98.4% in 2022 (*p *< 0.001). Overall, 21.3% (*n* = 27) of all patients did not undergo tumor resection due to the extent of vascular involvement (*n* = 11/127, 8.7%) or metastatic disease (*n* = 16/127, 12.6%). These latter 16 patients had either radiographic occult metastases detected during laparoscopy (*n* = 6/78, 7.8%) or during surgical exploration (*n* = 10/127, 7.8%). The remaining 100 patients (78.7%) underwent pancreatic resection, mostly pancreatoduodenectomy (*n* = 77/100, 77.0%). Vascular resections were performed in 68 patients (68.0%), including portomesenteric venous resection (*n* = 57/100, 57.0%) and/or arterial resection[s] (*n* = 17/100, 17.0%). Multivisceral resection was performed in nine patients (9.0%). Procedural details are summarized in Table 2. The resection rates for patients with NCCN LAPC (*n* = 36/49, 73.5%) at initial diagnosis did not differ from the resection rates in the remaining patients (*n* = 64/78, 82.1%; *p *= 0.250).

**Table﻿ 2:** Procedural details

Resected patients [*n* = 100]	
Diagnostic laparoscopy^a^, *n* (%)	78 (78.0)
Operation time, minutes, median [IQR]	332 [294–398]
Blood loss, mL, median [IQR]	550 [300–1015]
ASA-PS, *n* (%)	–
I–II	76 (76.8)
III–IV	23 (23.2)
Pancreatectomy, *n* (%)	–
Pancreatoduodenectomy	77 (77.0)
Distal pancreatectomy	17 (17.0)
Total pancreatectomy	6 (6.0)
Portomesenteric venous resection, *n* (%)	57 (57.0)
Type I–II	31 (54.4)
Type III–IV	26 (45.6)
Arterial resection, *n* (%)	17 (17.0)
Hepatic artery	6 (35.3)
Celiac axis	10 (58.8)
Superior mesenteric artery	1 (8.9)
Colectomy, *n* (%)	2 (2.0)
(Sub)total gastrectomy, *n* (%)	7 (7.0)
R1 resection, *n* (%)^b^	53 (53.0)

Non-therapeutic laparotomy [*n* = 27]	
Diagnostic laparoscopy, *n* (%)	23 (85.2)
Reason no resection, *n* (%)	–
Extensive local involvement	11 (40.7)
M1 detected by diagnostic laparoscopy	6 (22.2)
M1 detected by laparotomy	10 (37.0)
Palliative bypass surgery, *n* (%)	–
Hepaticojejunostomy	2 (7.4)
Gastro-enterostomy	1 (3.7)
Local ablative treatment, *n* (%)	–
Radiofrequency ablation	3 (11.1)
Irreversible electroporation	3 (11.1)

*n* Number of patients, *IQR* Interquartile range, *DLS* Diagnostic laparoscopy, *ASA-PS* American Society of Anaesthesiology Physical Status, *M1* Metastatic disease

^a^Missing data: operation time (*n *= 17), blood loss (*n *= 5), ASA-PS (*n *= 1)

^b^Definition is provided in Appendix 1

The rates of in-hospital/30-day mortality and major morbidity were 0.8% and 31.3%, respectively. No significant differences were seen for patients with or without resection in in-hospital/30-day mortality (1.0% vs. 0%; *p *= 0.999) and major morbidity (33.3% vs. 25.9%; *p *= 0.464), respectively. The 90-day mortality rate was 5.5%, which included 3.1% mortality due to early disease progression (in patients after resection this was 5.0% and 2.0%, respectively). Among patients who underwent resection, the R1 resection rate was 53.0% (*n* = 53) with pN0 resection in 36.0% (*n* = 36). In total, 61 patients (61.0%) received adjuvant therapy following resection (see Table 3 for details regarding surgical and histopathological outcome for both groups).

**Table 3:** Surgical outcome

		Total cohort		
Characteristics^a^	Overall [*n* = 127]	Resection [*n *= 100]	Non-therapeuticlaparotomy [*n* = 27]	*p*-Value
POPF, *n* (%)^b^				
Grade B	–	13 (13.8)	NA	–
Grade C	–	2 (2.1)	NA	–
Chyle leakage, *n* (%)	–			–
Grade B	–	24 (24.0)	NA	–
Grade C	–	9 (9.0)	NA	–
PPH, *n* (%)	–			–
Grade B	–	2 (2.0)	NA	–
Grade C	–	3 (3.0)	NA	–
Bile leakage, *n* (%)^c^	–			–
Grade B	–	7 (8.4)	NA	–
Grade C	–	1 (1.2)	NA	–
DGE, *n* (%)	–			
Grade B	–	5 (5.0)	NA	–
Grade C	–	8 (8.0)	NA	–
Major morbidity, *n* (%)	40 (31.3)	33 (33.3)	7 (25.9)	0.464
Relaparotomy	11 (8.7)	11 (11.0)	0 (0.0)	–
Organ failure	6 (4.7)	5 (5.0)	1 (3.7)	–
MCU/ICU admission	10 (7.9)	8 (8.0)	2 (7.4)	–
Adjuvant/palliative therapy^d^*, n* (%)	75 (59.1)	61 (61.0)	14 (51.9)	
Readmission, *n* (%)	25 (19.8)	22 (22.2)	3 (11.1)	0.207
Hospital stay, days median [IQR]	7 [5–13]	7 [6–14]	3 [2–4]	**0.001**
In-hospital/30-day mortality	1 (0.8)	1 (1.0)	0 (0.0)	>0.999
90-day mortality, *n* (%)	7 (5.5)	5 (5.0)	2 (7.4)	0.640
Complication related	3 (2.4)	3 (3.0)	0 (0.0)	–
Recurrence/progression related	4 (3.1)	2 (2.0)	2 (7.4)	–

Statistical significant variables only (P value < 0.05) are in bold

*n* Number of patients, *IQR* Interquartile range, *POPF* Postoperative pancreatic fistula, *PPH* Postpancreatectomy hemorrhage, *DGE* Delayed gastric emptying, *MCU/ICU* Medium/intensive care unit, *NA* Not available

^a^Missing data: POPF (*n *= 2), bile leakage (*n *= 4), major morbidity (*n *= 1), hospital stay (*n *= 1)

^b^Patients who underwent distal pancreatectomy were excluded from the nominator

^c^Patients who underwent total pancreatectomy were excluded from the nominator

^d^Patients undergoing resection received systemic adjuvant therapy and patients undergoing non-therapeutic laparotomy received systemic palliative therapy, as described in Appendix 1

^e^Patients who died during admission (i.e., in-hospital mortality) were excluded from the nominator

### Oncological Outcome

**Fig. 1:**
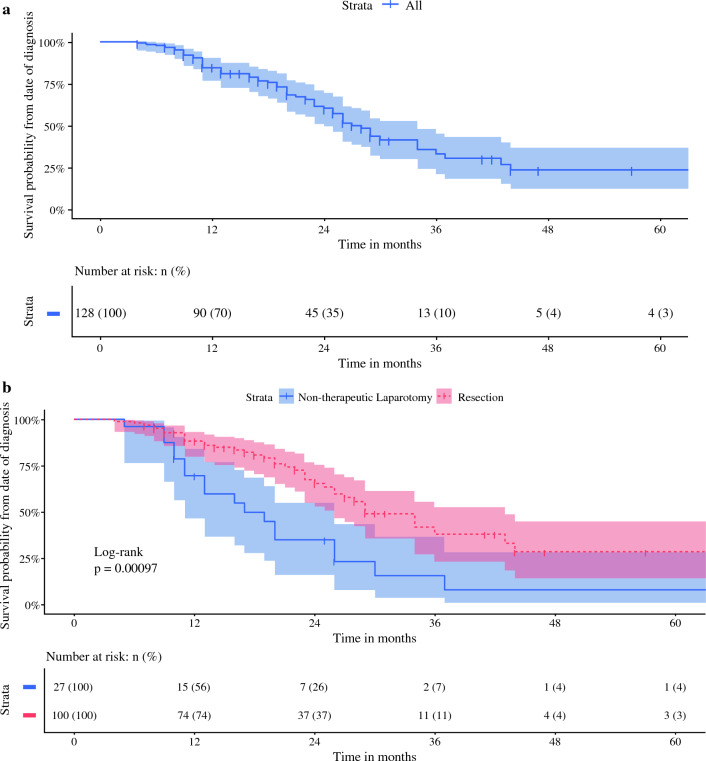
**a** Overall survival from diagnosis in the explored cohort (*n* = 127). **b** Overall survival from diagnosis resection (*n* = 100) versus non-therapeutic laparotomy (*n* = 27).

The median follow-up time from time of diagnosis in all patients was 27 months (IQR 22–29), whereby 70 patients (55.1%) were alive at the end of follow-up. The median OS was 28 months from the time of diagnosis (95% CI 24–34) and 23 months (95% CI 17-30) from the time of surgery. In the full cohort of 127 patients, the 1-, 3-, and 5- year OS rates from date of diagnosis were 84.6%, 33.2%, and 23.7%, respectively. Resection was associated with a longer median OS as compared with patients undergoing non-therapeutic laparotomy (29 months [95% CI 26-43] vs. 17 months [95% CI 11–26]*; p *< 0.001), as illustrated in Fig. 1. The 1-, 3-, and 5-year OS rates were 88.3%, 37.8%, and 28.4% in patients who underwent resection, respectively, versus 69.5%, 15.4%, and 7.7% in patients undergoing non-therapeutic laparotomy, respectively. The 5-year OS rates in patients with NCCN LAPC were 30.5% in those resected and 13.6% in those undergoing non-therapeutic laparotomy. In sensitivity analysis, all patients undergoing local ablative therapy (*n* = 6) were excluded from the non-therapeutic laparotomy group, resulting in a median OS of 14 months (95% CI 10–19) in the remaining 21 patients.

Disease recurrence was observed in 62% of patients after resection (*n* = 62/100). Most patients developed both locoregional recurrence and distant metastases (*n* = 31/64, 48.4%). Only local recurrence occurred in 18.8% (*n* = 12/64) and only distant metastases occurred in 30.6% (*n* = 19/64) of patients. The median recurrence-free survival was 10 months (95% CI 9–13), whereby 24 patients (24.0%) presented with recurrence at ≤6 months (early recurrence) and 21 patients (21.0%) presented with recurrence at ≥12 months (late recurrence).

In the overall cohort, Cox proportional hazard regression analysis only identified tumors located in the pancreatic body/tail as an independent negative predictor for OS (HR 1.788 [95% CI 1.042–3.068]) [see Table 4]. In patients undergoing resection, only the presence of positive lymph nodes at pathological assessment (ypN1-2) was identified as a negative predictor for OS (HR 3.503 [95% CI 1.630–7.527]) [see electronic supplementary Table 3 for the Cox regression analysis for resected patients].

**Table 4:** Cox proportional hazards regression analysis

Variables	Univariable analysis			Multivariable analysis		
	HR	95% CI	*p*-Value	HR	95% CI	*p*-Value
Age, years	1.011	0.960–1.020	0.484	–	–	–
ECOG-PS						
0–1	1 (reference)	–	–	–	–	–
≥2	0.848	0.264–2.723	0.782	–	–	–
NCCN LAPC				–	–	–
No	1 (reference)	–	–	–	–	–
Yes	0.885	0.664–1.924	0.652	–	–	–
Year of surgery				–	–	–
2014–2020	1 (reference)	–	–	–	–	–
2021–2022	0.597	0.287–1.242	0.168	–	–	–
Tumor location						
Head	1 (reference)	–	–	1 (reference)	–	–
Body/tail	1.788	1.042–3.068	**0.035**	1.788	1.042–3.068	**0.035**
Lymph node status at diagnosis						
cN0	1 (reference)	–	–	–	–	–
cN1–2	0.616	0.271–1.401	0.248	–	–	–
Missing	0.898	1.114–0.504	0.713	–	–	–
RECIST				–	–	–
Stable/progressive disease	1 (reference)	–	–	–	–	–
Partial/complete response	0.581	0.241–1.404	0.228	–	–	–
Missing	0.969	0.520–1.804	0.921	–	–	–
CA19-9 at restaging (U/mL)				–	–	–
<37 U/mL	1 (reference)	–	–	–	–	–
≥37 to <150 U/mL	0.624	0.308–1.264	0.190	–	–	–
≥150 U/mL	1.618	0.813–3.221	0.170	–	–	–
≥500 U/mL	1.108	0.439–2.796	0.828	–	–	–
CA19-9 response (%)						
Stable/increase	1 (reference)	–	–	–	–	–
<60% reduction	0.616	0.207–1.836	0.384	–	–	–
≥60% reduction	0.620	0.259–1.485	0.283	–	–	–
Missing	1.117	0.484–2.578	0.795	–	–	–
Preoperative therapy duration^a^				–	–	–
<4 months	1 (reference)	–	–	–	–	–
≥4 to <6 months	1.576	0.817–3.039	0.175	–	–	–
≥6 months	1.058	0.501–2.234	0.882	–	–	–

Statistical significant variables only (P value < 0.05) are in bold

*HR* Hazard ratio, *CI* Confidence interval, *ECOG PS* Eastern Cooperative Oncology Group performance status, *NCCN* National Comprehensive Cancer Network, *RECIST* Response valuation Criteria in Solid Tumors, *CA19-9* Carbohydrate antigen 19-9, *LAPC* Locally advanced pancreatic cancer

^a^Time from diagnosis to surgery was taken as a surrogate marker for preoperative therapy duration

### Trend in Surgical Exploration

The annual number of patients with LAPC undergoing surgical exploration increased over time from 6 in 2014 to 19 in 2022 (*p *= 0.543). No trend was seen in the number of non-therapeutic laparotomies over time (either absolute or relative to the total number of explorations), as outlined in electronic supplementary Fig. 1.

## Discussion

This third largest single-center experience demonstrated a 78.8% resection rate among 127 patients with LAPC selected for surgical exploration after induction chemotherapy. The in-hospital/30-day mortality was 0.8% and median OS after resection was 29 months. In the 27 patients in whom no resection was performed, 59.2% had occult metastatic disease. Median OS was significantly longer for patients undergoing resection, with a 5-year OS rate of 28.4% versus 7.7% in patients in whom no resection was performed. During the study period, the use of surgery for patients with LAPC gradually increased.

Previously, several high-volume centers from the United States, Germany, Sweden, Italy, France, and Japan have reported their experience with surgical resection for LAPC following induction chemotherapy.^11–17,29^ One study did not distinguish outcomes for patients with locally advanced or metastatic disease,^13^ and the remaining studies are summarized in Table 5. The majority of studies included both borderline resectable pancreatic cancer (BRPC) and LAPC as defined by the NCCN guideline,^11–17^ which is comparable with the DPCG definition for LAPC in the current study, as outlined in electronic supplementary Table 1. Of these, only one study previously reported on the surgical and oncological outcomes of all explored patients, and subsequently on outcomes of the non-therapeutic laparotomy group only.^29^ Herein, Michelakos and colleagues reported on 141 patients with BRPC/LAPC undergoing surgical exploration after (m)FOLFIRINOX with a similar resection rate (78.7% vs. 78.0%) and median OS after non-therapeutic laparotomy (17 vs. 19 months).^29^ However, potential predictors for OS in the total cohort were not investigated. A second study only reported on 3-year OS after non-therapeutic laparotomy (2.4%), which was considerably shorter than the 15.4% in the current study. The reasons for this difference are yet unclear.

**Table 5:** Overview single-center series on surgical exploration and resection in patients with LAPC

Study	Definition LAPC	Stage	Total cohort (*n*)	Resected (*%*)	mOS total explored^a^	mOS non-therapeutic laparotomy^a^	3-year OS non-therapeutic laparotomy (%)	mOS resected^a^	3-year OS resected (%)	30-day mortality resected (%)	90-day mortality Resected (%)	Predictors OS total cohort
Maggino et al.^14^	NCCN	BRPC/LAPC	147	93 (63.3)	–	–	–	35.4	–	–	–	–
Michelakos et al.^29^	AHPBA/SSO/ SSAT	BRPC/LAPC	141	110 (78.0)	34.2	18.6	–	37.7	–	1.4	–	–
Gemenetzis et al.^16^	NCCN	LAPC	116	84 (72.4)	–	–	–	35.3	50	1	4	–
Garnier et al.^17^	NCCN	BRPC/LAPC	–	98 (unknown)	–	–	–	39	–	6.1	8.2	–
Rangelova et al.^11^	NCCN	BRPC/LAPC	77	53 (68.4)	14.5	–	–	21.8	39.5	–	6	–
Truty et al.^12^^b^	NCCN	BRPC/LAPC	254	231 (90.9)	–	–	–	51.1	72	–	6.7	–
Ushida et al.^15^	NCCN	LAPC	123	20 (16)	–	–	–	NR	77	0	–	–
Current study (2023)	DPCG	LAPC	127	100 (78.7)	28	17	15.4	29	37.8	1.0	5.0	Yes

mOS median overall survival, NCCN National Comprehensive Cancer Network, AHPBA Americas Hepato-Pancreato-Biliary Association, SSO Society of Surgical Oncology, SSAT Society for Surgery of the Alimentary Tract *, mOS measured in months from diagnosis. Truty et al. (2021) reported mOS from time of surgery.

The remaining six studies only reported on outcomes in the resected patients. The in-hospital/30-day mortality and 90-day mortality rates after resection were comparable with those reported in the current study (0–6% vs. 1%)^13,15–17,29^ and (4–8% vs. 5%), respectively.^11,12,16,17,30^ Likewise, median OS from diagnosis (15–51 vs. 29 months)^11–13,16,17,29^ and actuarial 5-year OS (16–33% vs. 28%)^11,17,31^ following resection as described are in line with the current study.

Historically, there has been considerable reluctance towards surgical exploration in patients with LAPC.^32^ Despite 5-year OS rates reported up to 33%, recurrence occurs in more than 80% of patients and remains the principal cause of limited survival benefit following surgery.^33^ In particular, early recurrence (≤6 months) remains a major problem, as it occurs in one-third of patients after surgery for LAPC and is associated with decreased OS compared with patients with late recurrence (≥12 months) (8.4 vs. 27.4 months). This finding suggests the presence of occult systemic disease at the time of surgery.^34^ In this study, 62% of resected patients developed recurrence before the end of follow-up, associated with a median recurrence-free survival of 10 months. Additionally, approximately one-quarter of patients developed early recurrence, in whom surgery may not have been effective.

The inability to accurately predict patients at risk for (early) recurrence in combination with a non-therapeutic laparotomy rate of 21% underlines the persisting complexities in patient selection for surgery.^35^ Particularly in the current era of multiagent induction therapy, which is known to hamper the predictive value of CT-based restaging,^36^ the concept of tumor biology-driven response evaluation has become crucial in patient selection.^6^ This mainly relies on serum CA19-9 and, to a lesser extent, functional imaging modalities determining the metabolic and biological response to induction therapy.^37^ FDG-PET/CT could support decision making based on tumor markers as serum CA19-9, although more data are needed.^37^

In order to optimize patient stratification and surgical decision making in LAPC, numerous predictors for OS have been described, including sex, age, tumor diameter, vascular involvement, tumor markers, tumor differentiation, and length of preoperative therapy.^38–40^ Somewhat unexpected, besides tumor location, no preoperative predictors useful for patient selection were identified in the overall cohort in the current study. Serum CA19-9 levels at restaging, both as absolute values and categorized by response, were not identified as independent predictors for OS. This is likely due to the fact that patients included in the Cox regression analysis were, among others, selected to undergo surgical exploration based on biological response of CA19-9 following induction therapy. Predictors for OS described in the literature are often derived from an unselected cohort including all LAPC patients receiving systemic therapy, and hampered by the inclusion of tumor resection (yes/no) as an independent variable.^39,40^ A previous Dutch nationwide study identified age, sex, comorbidity status, and serum CA19-9 levels as independent predictors for OS, while only including preoperative parameters that may aid in patient selection.^38^ Nevertheless, the vast majority of the cohort comprised patients who did not undergo resection (*n* = 220/252). Among surgical candidates in patients with LAPC, predictors identified in an homogeneous cohort of patients with LAPC explored with curative intent may be of value in preoperative clinical decision making and, in particular, the prevention of non-therapeutic laparotomies and futile surgical resection by early disease recurrences. To the best of the authors knowledge, no study has reported on predictors identified in an overall cohort of patients undergoing surgical exploration for LAPC (i.e., with and without resection).

Besides preoperative selection, accurate intraoperative staging may prevent morbidity and loss of quality of life from futile operation. Nevertheless, 21% of explored patients underwent a non-therapeutic laparotomy in the current study, which is rather similar to the 28% reported by a recent multicenter study comprising five international expert centers, including our center.^10^ Diagnostic laparoscopy can aid in the detection of occult metastatic disease. In our center, patients undergoing surgical exploration for any stage of pancreatic ductal adenocarcinoma undergo laparoscopy in the same procedure as the intended laparotomy. However, lack of a standardized approach towards the use of laparoscopy in the early years of the study period resulted in only 79.9% of patients undergoing laparoscopy, which identified metastases in 7.8% of these patients. This is underlined by the increase seen in the use of laparoscopy from 16.7% in 2014 to 98.4% in 2022 (*p *< 0.001). Moreover, the reasons for incomplete laparoscopy (e.g. adhesions) were not registered and these data are therefore lacking. A previous retrospective study including 151 patients in whom occult metastatic disease was detected either by diagnostic laparoscopy (*n* = 89) or laparotomy (*n* = 62) found better OS if detected by laparoscopy (343 vs. 222 days), underlining its value.^41^ Interestingly, recent data from the North American National Surgical Quality Improvement Program (NSQIP) registry reported on a decreasing trend for the utilization of diagnostic laparoscopy in an 8-year study period, whereby only 10% of patients underwent staging laparoscopy (for all stages of localized pancreatic cancer).^42^ This is contradictory to the practice reported from several international expert centers and in the current study.^43–45^

In order to optimize oncological treatment strategies and subsequent patient selection for surgery in LAPC, the nationwide PREOPANC-4 trial was started in 2021 (ClinicalTrials.gov identifier: NCT05524090), implementing the international best practices for LAPC care in The Netherlands.^25^ In close collaboration with four international expert centers from the United States and Germany, multidisciplinary care and selection for surgery is currently being optimized by standardization of oncological treatments and patient selection for surgery. The rather similar 5-year OS rates in patients with NCCN LAPC compared with the overall cohort (30% vs. 28%) in combination with similar resection rates (73.5% vs. 82.1%; *p* = 0.250) illustrate the ongoing paradigm shift from anatomical-driven towards biology-driven patient selection for surgery. Future research should focus on improving response evaluation to induction therapy for surgery by the development of more personalized treatment and the identification and validation of new serological/imaging-based tumor markers. Molecular and genomic profiling could help to predict tumor sensitivity to induction therapy.^46,47^ Additionally, a wide spectrum of promising biomarkers have been described, such as inflammatory response parameters, circulating tumor DNA and microRNAs, which may help in patient selection.^48,49^ Nevertheless, it is important that their added value against CA19-9 is confirmed.^50^

## Conclusion

This high-volume, single-center analysis found a 78.7% resection rate in 127 selected patients with LAPC who underwent surgical exploration. Resection was associated with a median OS of 29 months and a 5-year OS rate of 28.4%, whereas the median OS and 5-year OS among 27 patients undergoing non-therapeutic laparotomy were 17 months and 7.7%, respectively. In the overall cohort, only tumor location was associated with OS. As predictors for OS to guide pre- and intraoperative decision making remain limited, future research should focus on optimizing the selection criteria for surgery in LAPC patients by identification and validation of tumor markers aimed at preventing futile operation.

### Supplementary Information

Below is the link to the electronic supplementary material.

Supplementary file1 (DOCX 131 KB)
