# Supplementary material for: Surgery for Locally Advanced Pancreatic Cancer Following Induction Chemotherapy: A Single-Center Experience
Source: Ann Surg Oncol. 2024 Jul 2;31(9):6180–92. doi: 10.1245/s10434-024-15591-4 (PMC11300483; doi:10.1245/s10434-024-15591-4)
Supplement: Supplementary file 1 — Supplementary file1 (DOCX 131 KB) [file 10434_2024_15591_MOESM1_ESM.docx]

**Supplementary Figure 1.** **Number of surgical explorations for LAPC over time**

**
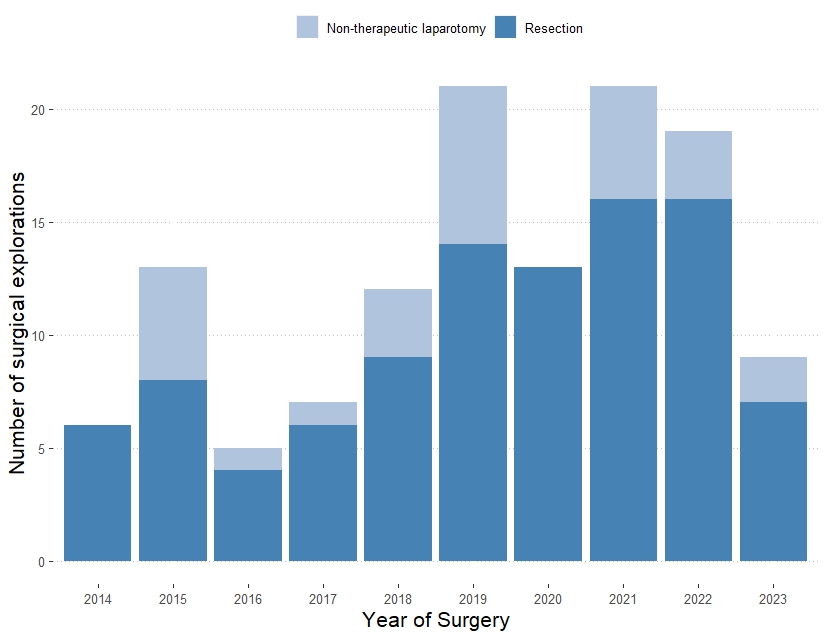
**

**Supplementary Table 1. LAPC definitions DPCG vs. NCCN**

| **SUPPLEMENTARY TABLE 1.** **Criteria for LAPC** | | | | |
| --- | --- | --- | --- | --- |
| **Vessel**  **Guideline** | **SMA** | **(C)HA** | **CA** | **SMV/PV** |
| **DPCG** | >90° | >90° | >90° | >270°/occlusion |
| **NCCN** | >180° | Non-reconstructable | >180° * | Non-reconstructable |
| *LAPC,* locally advanced pancreatic cancer; *SMA,* superior mesenteric artery; *(C)HA,* (common) hepatic artery; *CA,* celiac axis; *SMV,* superior mesenteric vein; *PV,* portal vein; *DPCG,* Dutch Pancreatic Cancer Group; *NCCN,* National Comprehensive Cancer Network.  *Borderline resectable pancreatic cancer if no involvement of the aorta and intact and uninvolved gastroduodenal artery (permitting a modified Appleby procedure). | | | | |

**Supplementary Table 2. Response to induction therapy**

| **SUPPLEMENTARY TABLE 2.** Response to induction therapy |  |  |  |  |  |  |
| --- | --- | --- | --- | --- | --- | --- |
|  |  |  |  |  |  |  |
|  |  |  | **TOTAL COHORT** | |  | |
| **Characteristics^*^** | **Overall** (*n* = 127) |  | **Resection** *(n* = 100) | **Non-therapeutic laparotomy** *(n* = 27) |  | ***P* value^** |
| RECIST^**^, *n* (%) | - |  | - | - |  | 0.191 ^c^ |
| Partial response | 24 (23.3) |  | 21 (27.3) | 3 (11.5) |  | - |
| Stable disease | 78 (75.7) |  | 54 (70.1) | 23 (88.5) |  | - |
| Progressive disease | 1 (1.0) |  | 2 (2.6) | 0 (0.0) |  | - |
| Missing | 24 |  | 23 | 1 |  | - |
| CA19-9 at restaging, median [IQR] | 62 (24 - 190) |  | 69 (28 - 178) | 36 (11 - 327) |  | 0.487 ^b^ |
| < 37 U/ml (normal) | 42 (35.3) |  | 28 (30.4) | 14 (51.9) |  | **0.004** ^c^ |
| ≥ 37 - < 150 U/ml | 40 (33.6) |  | 38 (41.3) | 2 (7.4) |  | - |
| ≥ 150 - < 500 U/ml | 25 (21.0) |  | 17 (18.5) | 8 (29.6) |  | - |
| ≥ 500 U/ml | 12 (10.1) |  | 9 (9.8) | 3 (11.1) |  | - |
| Missing | 8 |  | 8 | 0 |  | - |
| CA19-9 response, *n* (%) | - |  | - | - |  | 0.465 ^c^ |
| Normal to normal | 18 (17.8) |  | 12 (15.2) | 6 (27.3) |  | - |
| Elevated to normal | 16 (15.8) |  | 12 (15.2) | 4 (18.2) |  | - |
| Elevated to elevated | 64 (63.4) |  | 52 (65.8) | 12 (54.5) |  | - |
| Normal to elevated | 3 (3.0) |  | 3 (3.8) | 0 (0.0) |  | - |
| Missing | 26 |  | 21 | 5 |  | - |
| CA19-9 relative change, median [IQR]¶ | -72% (-86%-40%) |  | -42% (-81%-64%) | -76% (-87%-45%) |  | 0.069 ^b^ |
| Missing | 44 |  | 33 | 11 |  | - |
| CEA at restaging, median [IQR] | 4 (3 - 6) |  | 4 (3 - 6) | 4 (3 - 13) |  | 0.742 ^b^ |
| Normal (≤ 5 ng/ml), *n* (%) | 60 (63.2) |  | 48 (64.0) | 12 (60.0) |  | 0.682 ^a^ |
| Elevated (> 5 ng/ml), *n* (%) | 35 (36.8) |  | 27 (36.0) | 8 (40.0) |  | - |
| Missing | 32 |  | 25 | 7 |  |  |
| CEA dynamics, *n* (%) | - |  | - | - |  | 0.319 ^c^ |
| Normal to normal | 25 (52.1) |  | 23 (53.5) | 2 (40.0) |  | - |
| Elevated to normal | 9 (18.8) |  | 9 (20.9) | 0 (0.0) |  | - |
| Elevated to elevated | 9 (18.8) |  | 7 (16.3) | 2 (40.0) |  | - |
| Normal to elevated | 5 (10.4) |  | 4 (9.3) | 1 (20.0) |  | - |
| Missing | 79 |  | 57 | 22 |  |  |
| *RECIST* response evaluation criteria in solid tumors, *n* number of patients; *IQR* interquartile range; *CA19-9* carbohydrate antigen 19-9; *CEA* carcinoembryonic antigen;  ¶, patients with non-elevated CA19-9 at time of diagnosis are excluded from this analysis  ^ *a*, Pearson Chi square test; *b*, Mann-Whitney U test; *c* Fisher’s exact test | | | | | | |

**Supplementary table 3. Cox proportional regression analysis resection**

| **Supplementary Table 3. Cox proportional hazards regression analysis in patients undergoing resection** | | | | | | | |
| --- | --- | --- | --- | --- | --- | --- | --- |
|  | Univariable analysis | | |  | Multivariable analysis | | |
| **Variables*** | **HR** | **95%CI** | ***P* value** |  | **HR** | **95%CI** | ***P* value** |
| Age, years | 0.978 | 0.948-1.103 | 0.216 |  |  |  |  |
| ECOG-PS |  |  |  |  |  |  |  |
| 0-1 | 1 [reference] | - | - |  |  |  |  |
| ≥ 2 | 0.370 | 0.051-2.709 | 0.328 |  |  |  |  |
| NCCN LAPC |  |  |  |  |  |  |  |
| No | 1 [reference] | - | - |  |  |  |  |
| Yes | 1.233 | 0.644-2.361 | 0.527 |  |  |  |  |
| Year of surgery |  |  |  |  |  |  |  |
| 2014-2020 | 1 [reference] | - | - |  |  |  |  |
| 2021-2022 | 0.582 | 0.237-1.428 | 0.237 |  |  |  |  |
| Tumor location |  |  |  |  |  |  |  |
| Head | 1 [reference] | - | - |  |  |  |  |
| Body/tail | 1.633 | 0.826-3.232 | 0.159 |  |  |  |  |
| Lymph node status at diagnosis |  |  |  |  |  |  |  |
| cN0 | 1 [reference] | - | - |  |  |  |  |
| cN1-2 | 0.793 | 0.266-2.367 | 0.678 |  |  |  |  |
| Missing | 1.245 | 0.638-2.430 | 0.520 |  |  |  |  |
| RECIST |  |  |  |  |  |  |  |
| Stable/progressive disease | 1 [reference] | - | - |  |  |  |  |
| Partial/complete response | 0.642 | 0.234-1.764 | 0.391 |  |  |  |  |
| Missing | 1.157 | 0.864-0.572 | 0.685 |  |  |  |  |
| CA19-9 at restaging (U/ml) |  |  |  |  |  |  |  |
| < 37 U/ml | 1 [reference] | - | - |  |  |  |  |
| ≥ 37 - < 150 U/ml | 0.843 | 0.360-1.974 | 0.694 |  |  |  |  |
| ≥ 150 U/mL | 1.868 | 0.736-4.740 | 0.188 |  |  |  |  |
| ≥ 500 U/ml | 0.815 | 0.220-3.016 | 0.759 |  |  |  |  |
| CA19-9 response (%) |  |  |  |  |  |  |  |
| Stable/increase | 1 [reference] | - | - |  |  |  |  |
| < 60% reduction | 1.219 | 0.223-6.671 | 0.819 |  |  |  |  |
| ≥ 60% reduction | 1.657 | 0.382-7.185 | 0.500 |  |  |  |  |
| Missing | 2.258 | 0.518-9.847 | 0.278 |  |  |  |  |
| Preoperative therapy duration^¶^ |  |  |  |  |  |  |  |
| < 4 months | 1 [reference] | - | - |  |  |  |  |
| ≥ 4 to < 6 months | 1.556 | 0.721-3.360 | 0.260 |  |  |  |  |
| ≥ 6 months | 1.009 | 0.417-2.444 | 0.984 |  |  |  |  |
| Pancreatectomy |  |  |  |  |  |  |  |
| Pancreatoduodenectomy | 1 [reference] | - | - |  |  |  |  |
| Distal pancreatectomy | 1.161 | 0.480-2.808 | 0.740 |  |  |  |  |
| Total pancreatectomy | 2.703 | 1.035-7.058 | **0.042** |  |  |  |  |
| Portomesenteric vein resection |  |  |  |  |  |  |  |
| No | 1 [reference] | - | - |  |  |  |  |
| Type I-II | 0.783 | 0.349-1.759 | 0.554 |  |  |  |  |
| Type III-IV | 1.532 | 0.770-3.291 | 0.210 |  |  |  |  |
| Arterial resection |  |  |  |  |  |  |  |
| No | 1 [reference] | - | - |  |  |  |  |
| Yes | 1.571 | 0.646-3.823 | 0.319 |  |  |  |  |
| Residual disease |  |  |  |  |  |  |  |
| R0 | 1 [reference] | - | - |  |  |  |  |
| R1-2^#^ | 1.976 | 1.031-3.788 | **0.040** |  |  |  |  |
| Postoperative lymph node status |  |  |  |  |  |  |  |
| ypN0 | 1 [reference] | - | - |  | 1 [reference] | - | - |
| ypN1-2 | 3.503 | 1.630-7.527 | **0.001** |  | 3.503 | 1.630-7.527 | **0.001** |
| *HR* hazard ratio *95%CI* 95% confidence interval *ECOG PS* Eastern Cooperative Oncology Group performance status; *NCCN* National comprehensive Cancer Network; *CA19-9* carbohydrate antigen 19-9;  ^¶^, Time from diagnosis to surgery was taken as surrogate marker for preoperative therapy duration ^#^, No R2 disease occurred within this study cohort | | | | | | | |

**APPENDIX 1. Methodological definitions**

The 8^th^ edition of the TNM classification was used to classify the disease stage at time of diagnosis.^1^ The most proximal tumor location was registered when the tumor involved multiple anatomical locations were involved. Carbohydrate antigen 19-9 (CA19-9) and carcinoembryonic antigen (CEA) levels were considered elevated from ≥37 U/ml and ≥5 ng/ml, respectively. Radiological response after induction therapy was classified following the Response Evaluation Criteria in Solid Tumors (RECIST, version 1.1).^2^ The type and extent of surgery was defined in accordance with the International Study Group of Pancreatic Surgery (ISGPS).^3^ Complications were registered when they occurred during hospital stay or within 30 days after index surgery. Delayed gastric emptying (DGE), postpancreatectomy hemorrhage (PPH), bile leakage, postoperative pancreatic fistula (POPF), and chyle leakage were classified following the ISGPS and International Study Group of Liver Surgery (ISGLS) definitions.^4-8^ Residual disease (R status) in the resection margins was assessed and defined, following the Royal College of Pathologists whereby >1 mm margin clearance was defined as radical (R0). ^9^ Early recurrence (≤ 6 months) and late recurrence (≥12 months) were defined in accordance with Seelen et al.^10^ Adjuvant therapy was defined as the administration of at least one cycle of postoperative chemotherapy. Administration of any cancer-directed therapy (e.g. chemotherapy, immunotherapy, or radiotherapy) in patients who underwent non-therapeutic laparotomy was registered as palliative therapy.

Within the study period, two local ablative treatment modalities were in practice, namely radiofrequency ablation (RFA) and irreversible electroporation (IRE). RFA attempts to harm tumor tissue through thermal damage (i.e. increasing or decreasing temperatures sufficiently to induce cellular injury),^11^ whereas IRE uses high-voltage electrical pulses leading to necrotic and apoptotic cell death.^12^ Both treatments were solely given within the context of a clinical study: the Dutch multi-center randomized controlled trial assessing the efficacy of radiofrequency ablation (PELICAN trial; NCT03690323)^13^ and the prospective single-center cohort study assessing outcomes of IRE following induction therapy (IMPALA trial).^14^ Palliative bypass procedures comprised hepaticojejunostomy, gastro-enterostomy, or both, as relief and prevention of obstruction related symptoms are seen as a key component of the therapeutic management in the palliative setting.

**REFERENCES APPENDIX 1**

1. Kakar S. Protocol for the examination of specimens from patients with carcinoma of the pancreas. College of American Pathologists; 2017.

2. Eisenhauer EA, Therasse P, Bogaerts J, et al. New response evaluation criteria in solid tumours: revised RECIST guideline (version 1.1). Eur J Cancer. Jan 2009;45(2):228-47. doi:10.1016/j.ejca.2008.10.026

3. Hartwig W, Vollmer CM, Fingerhut A, et al. Extended pancreatectomy in pancreatic ductal adenocarcinoma: definition and consensus of the International Study Group for Pancreatic Surgery (ISGPS). Surgery. Jul 2014;156(1):1-14. doi:10.1016/j.surg.2014.02.009

4. Wente MN, Bassi C, Dervenis C, et al. Delayed gastric emptying (DGE) after pancreatic surgery: a suggested definition by the International Study Group of Pancreatic Surgery (ISGPS). Surgery. Nov 2007;142(5):761-8. doi:10.1016/j.surg.2007.05.005

5. Wente MN, Veit JA, Bassi C, et al. Postpancreatectomy hemorrhage (PPH): an International Study Group of Pancreatic Surgery (ISGPS) definition. Surgery. Jul 2007;142(1):20-5. doi:10.1016/j.surg.2007.02.001

6. Koch M, Garden OJ, Padbury R, et al. Bile leakage after hepatobiliary and pancreatic surgery: a definition and grading of severity by the International Study Group of Liver Surgery. Surgery. May 2011;149(5):680-8. doi:10.1016/j.surg.2010.12.002

7. Bassi C, Marchegiani G, Dervenis C, et al. The 2016 update of the International Study Group (ISGPS) definition and grading of postoperative pancreatic fistula: 11 Years After. Surgery. Mar 2017;161(3):584-591. doi:10.1016/j.surg.2016.11.014

8. Besselink MG, van Rijssen LB, Bassi C, et al. Definition and classification of chyle leak after pancreatic operation: A consensus statement by the International Study Group on Pancreatic Surgery. Surgery. Feb 2017;161(2):365-372. doi:10.1016/j.surg.2016.06.058

9. Campbell F. Dataset for the histopathological reporting of carcinoma of the pancreas, ampulla of Vater and common bile duct. The Royal College of Pathologists; 2017.

10. Seelen LWF, Doppenberg D, Stoop TF, et al. Minimum and Optimal CA19-9 Response After Two Months Induction Chemotherapy in Patients with Locally Advanced Pancreatic Cancer: A Nationwide Multicenter Study. Ann Surg. Jul 21 2023;doi:10.1097/sla.0000000000006021

11. Ahmed M, Brace CL, Lee FT, Jr., Goldberg SN. Principles of and advances in percutaneous ablation. Radiology. Feb 2011;258(2):351-69. doi:10.1148/radiol.10081634

12. Timmer FEF, Geboers B, Ruarus AH, et al. Irreversible Electroporation for Locally Advanced Pancreatic Cancer. Tech Vasc Interv Radiol. Jun 2020;23(2):100675. doi:10.1016/j.tvir.2020.100675

13. Walma MS, Rombouts SJ, Brada LJH, et al. Radiofrequency ablation and chemotherapy versus chemotherapy alone for locally advanced pancreatic cancer (PELICAN): study protocol for a randomized controlled trial. Trials. 2021;22(1):313.

14. Vogel JA, Rombouts SJ, de Rooij T, et al. Induction Chemotherapy Followed by Resection or Irreversible Electroporation in Locally Advanced Pancreatic Cancer (IMPALA): A Prospective Cohort Study. Ann Surg Oncol. Sep 2017;24(9):2734-2743. doi:10.1245/s10434-017-5900-9
